# Disability in Patients with Inflammatory Bowel Disease: Correlations with Quality of Life and Patient's Characteristics

**DOI:** 10.1155/2017/6138105

**Published:** 2017-05-29

**Authors:** Konstantinos Argyriou, Andreas Kapsoritakis, Konstantinos Oikonomou, Anastassios Manolakis, Eirini Tsakiridou, Spyridon Potamianos

**Affiliations:** Department of Gastroenterology, University Hospital of Larissa, Mezourlo 1, 41110 Larissa, Greece

## Abstract

**Background:**

Inflammatory bowel diseases may cause significant disability. However, little is known regarding the life domains where patients encounter most limitations.

**Objectives:**

To assess patients' overall disability and determine the life domains where most restrictions were applied. Secondarily, we sought for possible relationships among disability, quality of life (HRQoL), and population characteristics.

**Method:**

The study lasted for two years (2013–2015) and included 200 patients [52%  ulcerative  colitis  (UC)] from a referral centre. Disability was evaluated using the 36-item version of WHODAS 2.0 questionnaire. The influence of population characteristics on overall disability was assessed with linear regression.

**Results:**

Crohn's disease (CD) patients showed greater overall disability compared to UC (19.22 versus 15.01, *p* = 0.001), with higher scores in the domains of relationships, life activities, and participation. Disability was negatively associated with HRQoL (*p* < 0.001). Long activity, extensive disease, rural residence, and employment independently influenced the overall disability in both groups. Additionally, significant influence was recorded for lower education in the UC and for operation and celibacy in the CD group.

**Conclusions:**

CD patients were facing more limitations compared to those with UC, especially in the domains of relationships, activities, and participation. Other than clinical factors, sociodemographic characteristics were also associated with increased disability.

## 1. Introduction

Inflammatory bowel diseases (IBD), including mainly Crohn's disease (CD) and ulcerative colitis (UC), are chronic relapsing conditions of the gastrointestinal tract [1]. Over their longitudinal course, IBD may cause significant complications and disability to the patients that adversely affect their health related quality of life (HRQoL) [2, 3].

Contrary to progressive and irreversible medical conditions where disability is permanent and clinically recognizable, in relapsing diseases it may be temporary and difficult to address [4]. However, in all cases, disability increases the direct and the indirect cost of living with IBD [5]. Therefore, its prevention should be of particular concern among health care professionals and patient organizations [4, 5].

Until recently, the ability of patients to work was the most commonly used index in the assessment of IBD-related disability. However, working ability is not sufficient to fully cover the working limitations that patients may encounter during the long course of the intestinal disease; let alone the overall disability [5].

Although most disability studies report increased rates of unemployment, sick leave, and disability pension among IBD patients, most patients maintain their ability to work for many years after the initial diagnosis [5–7]. However, recent studies have entered new factors into play. IBD patients have been found to experience significantly more presenteeism (impaired productivity) and absenteeism (loss of working hours) compared to controls, with significant accompanying economic losses not only for the individual but also for the society [8, 9].

The latter shows that IBD represent a high economic burden to society. But, their cost could not be solely confined to economy [10].

Disability is an objective way of understanding the impact of IBD in patients' daily life. However, for many years, most studies were focusing on the subjective estimation of HRQoL when aiming to quantify the experience of living with these diseases [11]. A possible explanation for this shortage was probably the lack of standards not only in the assessment but also in the delimitation of the conceptual framework of disability.

In 2002, the World Health Organization (WHO) set the international standards for the assessment of disability. Including factors of activity and participation to employment as well as attitudes, WHO created the International Classification of Functioning, Disability and Health (ICF) [12].

Based on the concepts of ICF, generic and disease-specific instruments have been developed and become available to be used in the evaluation of disability in IBD [13–15]. Compared to disease-specific tools, generic questionnaires are offered for comparisons across different diseases and cultures. In addition, some generic measures, such as the WHO Disability Assessment Schedule 2.0 (WHODAS 2.0), allow the determination of the domains in patients' daily life where most functional limitations apply [15].

WHODAS 2.0 offers a multidimensional approach to disability. It is available in a short (12-item) and two longer (36-item/full and [12+24]-item/hybrid) versions, with the longer ones to provide additionally a detailed profile of disability. To date, WHODAS 2.0 has been tested in both inflammatory and noninflammatory conditions and has been found to be valid and reliable [15].

A recent study, using the short version of this questionnaire, has examined the prevalence of disability among IBD patients [16]. However, the profile of disability remained mostly unknown.

In our study, we used the 36-item version of WHODAS 2.0 in order not only to assess patients' overall disability but also to determine the domains of their daily life in which IBD patients experienced most restrictions. Secondary outcomes were to find possible relationships among disability, HRQoL, and population's characteristics.

## 2. Material and Methods

### 2.1. Patients and Design

The study took place at the University hospital of Larissa which constitutes the sole tertiary referral centre for IBD patients in Central Greece. The study was approved by the Ethics Committee and the Advisory Board of the University Hospital of Larissa (D.N.18484/17-04-2013). All participants provided consensus upon information about the nature, the duration, and the purpose of the study.

During a 2-year period (2013–2015), all consecutive IBD patients from our outpatient clinic and the gastroenterology department (where they have been admitted for an IBD-related cause) were screened for eligibility and entered the study.

Inclusion criteria were the patients to be adults, to be able to read and write Greek fluently, and to have IBD per standard criteria [17]. Patients diagnosed with a major neuropsychiatric disorder or other comorbidities prior to the diagnosis of the intestinal disease as well as those with stoma were excluded from the study, in order to avoid overestimation or underestimation in the assessment of HRQoL and disability.

The data were collected by the same investigator, who was not the treating doctor of the patient, during a personal interview that took place at a time specified by the patient in order to ensure the comfort and the accuracy of the answers. All interviews were held during the follow-up visit of the patients or within two days after admission.

Data collection included a diary card with the characteristics of the population and two measuring instruments for HRQoL and disability.

### 2.2. Population Characteristics

#### 2.2.1. Sociodemographic Variables

The background information included sex, age, place of residence, marital and employment status, and level of education for each patient.

#### 2.2.2. IBD-Related Variables

The clinical characteristics were as follows:

- type, duration, and extension of the intestinal disease,
- activity status (defined as short and long),
- smoking behaviour,
- disease behaviour (defined as complicated disease) in CD patients,
- history of previous IBD-related surgeries,
- previous hospitalizations,
- use of biologic agents > 3 months prior to study entry.

Disease activity was assessed by using the clinical indices Harvey-Bradshaw (HB) for CD and Simple Clinical Colitis Activity Index (SCCAI) for UC patients [18, 19]. Short activity referred to the patient's activity status at the time of recruitment. Long active was considered the disease for which activity had been recorded in most of the follow-up visits of the patient over the previous 3 years, based on patient's own medical record. Regarding activity, IBD patients were divided into two categories. Values < 5 in both indices were considered to indicate clinical remission.

### 2.3. Measuring Instruments

#### 2.3.1. Disability

For the assessment of disability, we used the interviewer-administered 36-item version of the questionnaire WHODAS 2.0. There are two other versions of WHODAS- the brief (12-item) and the hybrid (12+24-item). However, the 36-item (full) version is regarded the most detailed one.

WHODAS 2.0 is a practical, generic tool that can measure health and disability at population level or in clinical practice, without targeting a specific disease. It is available in 14 languages, including the Greek language. Its psychometric properties were subjected to two waves of international testing with the instrument to have been proved valid and reliable.

The full version has 36 questions that relate to the functioning difficulties experienced by the individual during the previous 30 days and captures disability in the following six domains:

1. Cognition: understanding and communication (6 questions).
2. Mobility: moving and getting around (5 questions).
3. Self-care: attending to one's hygiene, dressing, eating, and staying alone (4 questions).
4. Getting along: interpersonal relationships (5 questions).
5. Life activities: domestic responsibilities, work, and school (8 questions).
6. Participation: joining in community activities and social participation (8 questions).

In addition, the final part of the questionnaire includes an optional set of questions in which the responder is called to attribute the difficulties to a particular causative agent and to estimate their impact on his/her everyday life.

The answer to each question follows a 5-point Likert scale with the lowest score to show the smallest difficulty. The total and dimensional scores range from 0 to 100 with the highest score to show greater disability. There are two different ways to compute WHODAS 2.0 summary scores: the simple scoring option, which simply summarises the scores of the items across all domains, and the more complex scoring method, which is called “item-response-theory” (IRT) based scoring. Since simple scoring “is specific to the sample at hand and should not be assumed to be comparable across populations,” in our study, we used the complex scoring system which allows a more fine-grained analysis across populations or subpopulations. In complex scoring, summary scores result from standardized SPSS (SYNTAX) algorithms [15].

#### 2.3.2. HRQoL

For the assessment of HRQoL, we used the Greek version of the Inflammatory Bowel Disease Questionnaire (IBDQ) [20]. IBDQ has been widely used in clinical and epidemiological studies [21, 22].

### 2.4. Statistical Analysis

The study population was divided into two groups according to disease type. Statistical comparisons between the 2 groups were made either with Pearson's chi-square test (for categorical variables), or Mann-Whitney-Wilcoxon test (for continuous variables). Most summary scores in WHODAS 2.0 and IBDQ did not follow the normal distribution. Comparisons between continuous variables were performed with Spearman correlation coefficients. Possible relationships between disability and population's characteristics were evaluated with Mann-Whitney-Wilcoxon test for 2 groups and Kruskal-Wallis test when comparisons involved more than 2 groups.

Factors significantly associated with WHODAS 2.0 summary scores were entered into a linear regression analysis that was performed stepwise (backward elimination of variables).

The level of statistical significance was set at *p* < 0.05. For the statistical analysis, we used the statistical software SPSS 17 for windows.

## 3. Results

Over the study period, 253 patients were eligible to participate. From the initial population, 33 patients did not wish to participate (due to lack of time) and 20 patients were excluded because of inconclusive answers. Thus, the final population of the study consisted of 200 patients (52% with UC). The majority of the patients (86.5%) were recruited from our IBD clinic while 13.5% were hospitalized.

The sociodemographic and clinical characteristics of the final population are shown in Table 1. As for the population characteristics, no statistical significant differences were found between the excluded patients and the final population.

### 3.1. Profile and Measurement of Disability among IBD Patients

The majority of IBD patients (61.5% versus 67.7% for those with UC and CD, resp.) replied to the disability questions by comparing their current health status with the period that preceded the appearance of the intestinal disease.

Table 2 shows the mean total and dimensional WHODAS scores of the study population per disease type.

In general, CD patients scored higher compared to those with UC. Apart from cognition, mobility, and self-care, this difference was statistically significant in all other domains. The highest scores were achieved in the domains of relationships, life activities, and participation by both groups.

### 3.2. Per Domain Analysis

Over the 30 day reference period, the mean number of days that IBD patients had to cut down their usual daily activities was 9.26 ± 4.99 for the UC and 13,1 ± 6.53 for the CD group, respectively (*p* = 0,001).

#### 3.2.1. Cognition

In the domain of understanding and communication, 48% of IBD patients estimated that they had experienced difficulties in finding solutions to daily problems.

Between the 2 groups, CD patients encountered difficulties in launching and maintaining a conversation more frequently (33.7% versus 47.9% for UC and CD, resp., *p* = 0,041).

No statistically significant differences were found regarding the other domain specific items.

#### 3.2.2. Mobility and Self-Care

No statistically significant differences were found in the domains of mobility and self-care, with the majority (>80%) of IBD patients not reporting any specific restriction.

#### 3.2.3. Getting Along

In the domain of interpersonal relationships, irrespectively of the disease type, more than 70% of the patients were facing difficulties in making new friends and in intimate relationships.

Additionally, the majority of the population experienced functional limitations in sexual life (67.7% for CD versus 64.4% for UC, *p* > 0.05).

Between the 2 groups, CD patients had limitations in getting along with their friends more frequently (76% versus 59.6%, *p* = 0,014).

#### 3.2.4. Life Activities

Difficulties in performing the usual work-tasks were encountered by 73.6% of the participants with 55.7% of IBD patients having experienced difficulties in completing all the jobs of their daily schedule.

The majority of IBD patients (>60%) reported that they had worked at a slow pace with those suffering from CD having lost more profits (50% versus 33%, *p* = 0.046).

Similar restrictions applied in the subdomain of domestic activities. Approximately, half of the study population (56% for the CD and 52.8% for the UC group, resp.) encountered functional limitations in the completion of the household tasks.

In addition, compared to the patients with UC, those with CD estimated that they needed more time in order to perform their domestic activities (68.7% versus 37.5%, *p* = 0.001).

#### 3.2.5. Participation

In the domain of participation, difficulties were encountered by the vast majority of the population (>90%).

69.5% of the IBD patients estimated that they had been emotionally affected by their intestinal disease, with a similar proportion of patients (68%) spending time on their health condition and having difficulty in doing things for leisure or pleasure.

Although more than 50% of the UC patients reported to have encountered functional limitation in social participation because of barriers or hindrances from the other people around them, this problem was more prominent among CD patients (52.9% versus 72.9% for UC and CD, resp., *p* = 0.004). Because of others' attitudes, the same group of patients faced also a higher problem of decent living (71% for CD versus 45% for UC, *p* = 0.001).

Between the two groups, a similar proportion of patients perceived IBD as a financial (42.3% for UC versus 47.9% for CD) or as a family problem (40.4% for UC versus 40.6% for CD).

### 3.3. Impact and Severity of Functional Limitations in Daily Life

The impact of the functional limitations on patients' daily life was rated “moderate” by the majority of the study population (62%).

Across domains, the severity of the restrictions was ranging from mild to moderate for the majority of the responders in both patient groups.

### 3.4. Associations of Disability with HRQoL and Study Variables

The median IBDQ score was 172 and 179.5 for CD and UC patients, with the difference being statistically significant (*p* = 0.02)

Moderate to high negative correlations were found for all summary scores between WHODAS 2.0 and IBDQ (*p* < 0.001).

### 3.5. Multivariate Analysis

The relationships between disability and population characteristics were examined with univariate analysis. The results are shown in Tables 3 and 4.

All factors significantly associated with disability were entered into linear regression. The results are shown in Tables 5 and 6.

Extensive (defined as disease location beyond the splenic flexure) disease, long activity, rural residence, and full-time employment had an independent influence on the sum score of overall disability in both types of intestinal disease.

Additionally, a statistically significant association with increased disability was found for lower (defined as basic/secondary) education in patients with UC as well as for operations and celibacy (defined as single/divorced) in the CD group.

## 4. Discussion

In this project, our primary aims were not only the assessment of the IBD-related disability, but also the determination of the profile of the functional limitations that IBD patients experience in their daily life. Secondary outcomes were to search for possible relationships among disability, quality of life, and patients' characteristics. To our knowledge, this is the first study that highlights specific areas of functioning among IBD patients. In Greece, in particular, no previous studies described an individual's profile of the restrictions in functionality in sickness or in health with the 36-item version of WHODAS.

Power analysis indicated that, in order to ensure a power of 0.90 for coefficients as low as 0.25, we needed a total sample size of 164 participants. For comparisons between disease types, we needed at least 200 patients. In order to reduce measurement error, we used highly valid outcome measures.

Our data analysis showed that the level of disability in our population was higher than the one reported in a recent Canadian study [16]. This difference could be attributed to the different characteristics of the study population. In accordance with the same investigators, we observed that CD patients had a higher level of disability compared to those with UC. However, a small to medium effect size was found in the comparisons of all WHODAS 2.0 summary scores between the two patient groups.

The greater restrictions in our patients were recorded in the domains of interpersonal relationships and life activities as well as in the domain of social participation.

Qualitative studies from Europe [23], Australia [24], and Canada [25] suggested that living with IBD affects negatively the interpersonal relationships and the HRQoL of patients. Our findings support these studies. However, contrary to Becker et al. [25], who found that the impact of the intestinal disease on interpersonal relationships is severe, in our study, we found this impact to be mild to moderate for the majority of the population. One possible explanation for this finding is that the Greek IBD patients have strong family support which attenuates the burden of the disease [26]. Therefore, it may indicate how important it is for IBD patients to have a supportive environment in order to cope with the chronic disease.

In the domain of interpersonal relationships, the majority of our population has experienced limitations in making new friends, maintaining a friendship and in sex life. Friendship and sexuality can be both negatively affected in IBD with an accompanying negative impact on patients' physical and psychosocial well-being [27, 28]. Patient support groups could be of importance so as to reduce these limitations and improve patients' functionality.

Apart from relationships, IBD interfere with patients' working ability [29–33]. In our study, despite the fact that 85% of the participants maintained their working capacity, the majority admitted having worked at a slow pace and having difficulties in meeting all work commitments with subsequent loss of profits. Our findings are in line with the results of a recent study from the USA which showed increased presenteeism among IBD patients [8]. Presenteeism is an important issue for IBD patients, not only because it highlights the increased indirect cost of living with these diseases, but also because it may reflect patients' ability to cope with the working limitations that are imposed by the intestinal disease [8]. Therefore, its prevention should be of particular concern among health care professionals and organizations in order not only to increase productivity, but also to help our patients adjust to their disease.

Other than productivity at work, similar restrictions of functionality were applied in the performance of domestic tasks, indicating the additional burden that the caregivers often have to bear so as not to interrupt family functioning [34].

In the domain of participation, social activities and leisure have been found to be compromised in our population. These findings are in accordance with other studies [25, 35]. Similarly to our findings, Becker et al. [25] reported a higher impact of IBD on CD patients, attributing the difference to a more pronounced psychological morbidity. However, in our study, the emotional impact of IBD has not been found to differ between groups. A plausible explanation of our findings is that the majority of our patients encountered problems because of barriers and attitudes from the world around them, emphasizing the importance of the environment in the assessment and interpretation of disability.

In all domains of functionality, the severity of the experienced limitations was mild to moderate for the majority of the population. However, these limitations strongly affected patients' quality of life.

In the present study, we found that CD patients had worse quality of life compared to those with UC, with our finding being in accordance with the results of a recent Cypriot study [22]. Taking into account the fact that Greece and Cyprus have the same linguistic but different cultural characteristics, it may be of interest to see whether Cypriot patients experience similar limitations as well.

In regard to the predisposing factors for increased disability in our population, statistically significant associations were found for extensive or long active disease, rural residence, and full-time employment in both patient groups. In addition, statistically significant influence was recorded for lower education in the UC and for operation and marital status in the CD group. Concerning activity, the relationship with disability is easily understandable and not surprising [36, 37]. But for the other parameters, we have not detected corresponding references in literature.

Surprisingly enough, we found that full-time employment was independently associated with increased disability. This finding is opposite to other disability studies [30, 31]. However, it may indicate an attenuated ability of our population to deal with common work-related problems (e.g., fatigue, decreased motivation, and competitiveness) which, in turn, may lead the patients to cut down on their activities and/or to enlarge the restrictions in the other domains of their daily life. Consequently, empowering patients' ability to fight common work-related problems could help IBD patients to improve their overall functionality and/or to prevent disability.

The strength of this study is the use of the full version of the questionnaire WHODAS 2.0. Apart from a multidimensional approach of disability, this version allows researchers not only to make comparisons across different populations and cultures, but also to determine a profile of the functional limitations that an individual experience in his/her daily life.

Considering the potential limitations, we should note the inclusion of patients from a tertiary referral centre. Patients from referral centres are expected to have more severe disease. However, the absence of another IBD-specific healthcare provider in Central Greece has allowed the collection and presentation of data from the majority of IBD patients from our geographical area. Nevertheless, the size of our population was relatively small which precluded us from further subgroup analysis (e.g., to find differences between operated and nonoperated patients or between different treatments) or generalization.

Another potential limitation is the cross-sectional character of our study. However, this type of study allowed us the collection of information about the burden of the intestinal disease several years after the diagnosis and suggested associations that could be used for comparisons in longitudinal studies.

Finally, we note that, in our study, we used a general measurement tool for disability instead of the newly developed disease-specific tools. Apart from the fact that the IBD-specific tools were developed later in the course of our study, their results are not offered for comparisons across different populations; let alone for determining a profile of the functional limitations of IBD patients which was our primary goal. Nevertheless, we believe that a combined approach of overall disability in the future would help researchers to understand in full the various aspects of living with IBD, allowing an evidence-based patient-centred approach in the management of IBD patients.

## 5. Conclusions

Taking into account the possible limitations of this study, CD patients were found to experience more restrictions in their daily life compared to those with UC, especially in the domains of relationships, activities, and participation. Other than disease related factors, sociodemographic characteristics were found to negatively influence patients' overall disability. Determining the profile of the functional limitations of IBD patients may be of specific value for health care providers and organizations when organizing interventions that aim either to improve or to prevent IBD-related disability.

## Figures and Tables

**Table 1:** Population characteristics.

	Crohn disease (*N* = 96)	Ulcerative colitis (*N* = 104)	
*Sex*			
Male	46 (47.9)	51 (49)	NS
Females	50 (52.1)	53 (51)	
*Age (mean* ± *sd)*	39,2 ± 11.88	42.1 ± 12.9	NS
*Residence*			
Rural (<4.000 inhabitants)	25 (26)	32 (30.8)	
Semirural (4.000–10.000)	23 (24)	21 (20.2)	NS
Urban (>10.000)	48 (50)	51 (49.0)	
*Level of education*			
Basic	12 (12.50)	12 (11.5)	
Secondary	52 (54.17)	50 (48.1)	NS
Postsecondary	32 (33.30)	42 (40.4)	
*Marital status*			
Single	25 (26.1)	40 (38.5)	
Married	63 (65.6)	50 (48.1)	*p* = 0.012
Divorced/widowed	8 (08,3)	14 (13.4)	
*Employment status*			
Unemployed	20 (20.8)	20 (19.2)	
Part-time employed	21 (21,9)	22 (21.2)	NS
Full-time employed	47 (49.0)	50 (48.1)	
Pensioner	8 (08.3)	12 (11.5)	
*Active smoking*			
Yes	34 (35.4)	27 (26)	NS
No	62 (64.6)	77 (74)	
*Disease duration (mean ± sd in years)*	9.11 ± 2.041	9.47 ± 5.14	NS
*Short disease activity*			
Remission	30 (31.3)	37 (35.6)	
Active	66 (68.7)	67 (64.4)	NS
*Long disease activity*			
No	68 (70.8)	74 (71.2)	NS
Yes	28 (29.2)	30 (28.8)	
*Biologic agents*			
No	76 (79.2)	92 (88.5)	NS
Yes	20 (20.8)	12 (11.5)	
*IBD-related surgery*			
No	82 (85.4)	99 (95.2)	*p* = 0.021
Yes	14 (14.6)	5 (4.8)	
*IBD-related previous hospitalization*			
No	78 (81.3)	92 (88.5)	NS
Yes	18 (18.8)	12 (11.5)	
*Disease location*			
*Ulcerative colitis*			
Proctitis	NA	10 (9.6)	
Up to splenic flexure	NA	35 (33.7)	
Beyond splenic flexure	NA	54 (51.9)	
Pouch	NA	5 (4.8)	
*Crohn's disease*			
Colon	6 (6.3)	NA	
Ileum	55 (57.3)	NA	
Ileocolonic	35 (36.5)	NA	
*Complicated disease*		NA	
Inflammatory	60 (62.5)	NA	
Noninflammatory (stenotic-fistulizing-abscessing)	36 (37.5)	NA	

NA: non applicable; NS: non significant; *p* < 0.05 statistically significant.

**Table 2:** Comparison of the total and dimensional summary scores for WHODAS 2.0 between the two patients' groups.

	Ulcerative colitis (*n* = 104)	Crohn's disease (*n* = 96)	Cohen's *r*^*∗*^	*p* ^*∗∗*^
	Mean/median (range)	Mean/median (range)		
Cognition	6.64/5 (25)	8.59/5 (30)	NC^*∗∗∗*^	NS^*∗∗∗∗*^
Mobility	3.91/0 (37.50)	4.23/0 (43.75)	NC	NS
Self-care	2.98/0 (40)	3.85/0 (40)	NC	NS
Relationships	29.09/33.33 (58.33)	35.59/41.67 (58.33)	0.26	<0.001
Life activities (domestic tasks)	19.04/20 (90)	25.31/30 (90)	0.20	0.005
Life activities (work/school tasks)	20.24/21.43 (78.57)	26.47/28.57 (78.57)	0.18	<0.001
Participation	24/25 (54.17)	29.95/29.17 (58.33)	0.29	0.030
Overall disability (total score)	15/15.01 (36.79)	18.94/19.22 (40.57)	0.22	0.001

^*∗*^ *r* = 0.1, 0.3, and 0.5 for small, medium, and large effect size; ^*∗∗*^*p* < 0.05: statistical significance; ^*∗∗∗*^NC: not calculated; ^*∗∗∗∗*^NS: no statistical significance.

**Table 3:** Relationships between WHODAS 2.0 summary scores and population's characteristics in patients with Crohn's disease.

	WHODAS 2.0							
	Cognition *z*/*x*/*r* *p*	Mobility *z*/*x*/*r* *p*	Self-care *z*/*x*/*r* *p*	Relationships *z*/*x*/*r* *p*	Life activities (domestic tasks) *z*/*x*/*r* *p*	Life activities (work) *z*/*x*/*r* *p*	Participation *z*/*x*/*r* *p*	Total score *z*/*x*/*r* *p*
*Population* *characteristics*								
Sex	NS	NS	NS	NS	NS	NS	NS	NS
Age	NS	NS	NS	NS	NS	NS	NS	NS
Residence	9.768 0.008	NS	NS	10.815 0.004	6.701 0.035	6.679 0.035	13.876 0.001	14.143 0.001
Education	NS	NS	NS	NS	NS	NS	NS	NS
Marital status	8.155 0.017	NS	NS	10.284 0.006	8.279 0.016	8.143 0.017	10.493 0.005	13,944 0.001
Employment status	21.285 0.001	NS	NS	15.330 0.002	23.843 0.001	8.331 0.040	NS	18.314 0.001
Smoking	NS	NS	NS	NS	NS	NS	NS	NS
Disease duration	NS	NS	NS	NS	NS	NS	NS	NS
Short activity	NS	−2.194 0.028	−2.056 0.040	−3.039 0.002	−2.554 0.011	−2.134 0.033	−2.818 0.005	−3.318 0.001
Long activity	−4.405 0.001	−3.953 0.001	−2.387 0.017	−5.058 0.001	−5.798 0.001	−5.460 0.001	−5.338 0.001	−6.965 0.001
Biologic agents	NS	NS	NS	NS	NS	NS	NS	NS
IBD-related surgery	NS	−4.091 0.001	−2.898 0.004	−2.833 0.005	−3.986 0.001	−2.152 0.031	−3.864 0.001	−4.082 0.001
Previous hospitalization	NS	−4.489 0.001	−3.412 0.001	NS	−2.956 0.003	−3.046 0.002	−3.195 0.001	−3.330 0.001
Disease-location	11.534 0.003	NS	NS	13.972 0.001	NS	NS	8.028 0.018	8,728 0.013
Complicated disease	NS	NS	NS	−1.991 0.046	NS	NS	−2.168 0.030	NS

NS: non statistically significant; *p* < 0.05: statistically significant; *z*/*x*/*r*: Mann-Whitney-Wilcoxon test or Kruskal-Wallis test or Spearman correlation coefficients.

**Table 4:** Relationships between WHODAS 2.0 and population's characteristics in patients with ulcerative colitis.

	WHODAS 2.0							
	Cognition *z*/*x*/*r* *p*	Mobility *z*/*x*/*r* *p*	Self-care *z*/*x*/*r* *p*	Relationships *z*/*x*/*r* *p*	Life activities (domestic tasks) *z*/*x*/*r* *p*	Life activities (work) *z*/*x*/*r* *p*	Participation *z*/*x*/*r* *p*	Total score *z*/*x*/*r* *p*
*Population* *characteristics*								
Sex	NS	NS	NS	NS	NS	−2.287 0.022	−3.051 0.002	−1.972 0.049
Age	NS	NS	NS	NS	NS	NS	NS	NS
Residence	22.756 0.001	NS	NS	16.288 0.001	12.140 0.002	22.518 0.001	26.318 0.001	25.423 0.001
Education	34.481 0.001	NS	10.312 0.006	30.931 0.001	30.819 0.001	20.511 0.001	21.166 0.001	45.348 0.001
Marital status	21.411 0.001	NS	NS	20.290 0.001	16.652 0.001	14.002 0.001	20.434 0.001	24.073 0.001
Employment status	14.527 0.002	NS	NS	16.075 0.001	16.505 0.001	11.943 0.008	19.037 0.001	24.022 0.001
Smoking	NS	NS	NS	NS	NS	NS	NS	NS
Disease duration	0.310 0.001	NS	NS	0.256 0.009	0.276 0.005	0.256 0.030	0.281 0.004	0.302 0.002
Short activity	−3.698 0.001	NS	NS	−3.954 0.001	−4.061 0.001	−2.386 0.017	−3.717 0.001	−4.037 0.001
Long activity	−2.516 0.012	−4.503 0.001	−2.632 0.008	−3.411 0.001	−3.495 0.001	−3.729 0.001	−4.497 0.001	−4.787 0.001
Biologic agents	NS	−2.673 0.008	NS	NS	NS	NS	NS	NS
IBD-related surgery	−2.574 0.010	NS	NS	−3.468 0.001	−2.770 0.006	−3.052 0.002	−3.323 0.001	−3.475 0.001
Previous hospitalization	NS	−2.085 0.037	NS	NS	NS	−2.059 0.039	NS	−2.164 0.030
Disease-location	22.428 0.001	NS	NS	36.132 0.001	24.567 0.001	9.240 0.026	24.774 0.001	29.824 0.001

NS: nonstatistically significant; *p* < 0.05: statistically significant; *z*/*x*/*r*: Mann-Whitney-Wilcoxon test or Kruskal-Wallis test or Spearman correlation coefficients.

**Table 5:** Predictors for increased overall disability among population characteristics on the basis of multiple linear regressions in patients with Crohn's disease.

	Adjusted *R* square	Unstandardized coefficients				95% confidence interval for *B*		Collinearity statistics
		*B*	Std Error	*t*	*p*	Lower bound	Upper bound	VIF
Long activity		9.512	1.410	6.744	0.001	6.709	12.314	1.493
IBD-related operations		6.168	1.767	3.491	0.001	2.658	9.679	1.413
Extensive disease (colitis)	0.703	−7.934	2.230	−3.558	0.001	−12.364	−3.503	1.058
Place of residence (rural)		3.326	1.336	2.490	0.015	0.672	5.981	1.249
Marital status (celibacy)		3.083	1.173	2.629	0.01	0.752	5.414	1.128
Employment status (full-time job)		5.530	1.140	4.850	0.001	3.265	7.796	1.181

*p* < 0.05: statistically significant.

**Table 6:** Predictors for increased overall disability among population characteristics on the basis of multiple linear regression in patients with ulcerative colitis.

	Adjusted *R* square	Unstandardized coefficients				95% confidence interval for *B*		Collinearity statistics
		*B*	Std Error	*t*	*p*	Lower bound	Upper bound	VIF
Long activity		6.430	1.065	6.036	0.001	4.316	8.545	1.212
Education		6.298	1.126	−5.591	0.001	−8.534	−4.062	1.589
Extensive disease (beyond splenic flexure)	0.700	3.023	1.093	2.767	0.007	0.854	5.192	1.550
Place of residence (rural)		3.894	1.063	3.663	0.001	1.784	6.003	1.252
Marital status (celibacy)		NS	NS	NS	NS	NS	NS	NS
Employment status (full-time job)		2.204	0.954	2.311	0.023	0.311	4.097	1.212

NS: nonstatistically significant; *p* < 0.05: statistically significant.

## References

[1] Bannaga A. S., Selinger C. P. (2015). Inflammatory bowel disease and anxiety: links, risks, and challenges faced. *Clinical and Experimental Gastroenterology*.

[2] Lönnfors S., Vermeire S., Greco M., Hommes D., Bell C., Avedano L. (2014). IBD and health-related quality of life—discovering the true impact. *Journal of Crohn's and Colitis*.

[3] Colombel J.-F. (2013). Measuring disability in IBD: the IBD disability index. *Gastroenterology & Hepatology*.

[4] Bernklev T., Jahnsen J., Henriksen M. (2006). Relationship between sick leave, unemployment, disability, and health-related quality of life in patients with inflammatory bowel disease. *Inflammatory Bowel Diseases*.

[5] Abraham B. P., Sellin J. H. (2012). Disability in inflammatory bowel disease. *Gastroenterology Clinics of North America*.

[6] Ramos A., Calvet X., Sicilia B. (2015). IBD-related work disability in the Community: prevalence, severity and predictive factors. A cross-sectional study. *United European Gastroenterology Journal*.

[7] van der Valk M. E., Mangen M.-J. J., Leenders M. (2014). Risk factors of work disability in patients with inflammatory bowel disease—a Dutch nationwide web-based survey. Work disability in inflammatory bowel disease. *Journal of Crohn's and Colitis*.

[8] Zand A., Van Deen W. K., Inserra E. K. (2015). Presenteeism in inflammatory bowel diseases: a hidden problem with significant economic impact. *Inflammatory Bowel Diseases*.

[9] Schwartz D., Greenberg D., Chernin E. (2016). P592. Absenteeism and presenteeism amongst Crohn’s disease patients: results from a real-world cohort in Israel. *Journal of Crohn's and Colitis*.

[10] Rubin D. T., Siegel C. A., Kane S. V. (2009). Impact of ulcerative colitis from patients' and physicians' perspectives: results from the UC: NORMAL survey. *Inflammatory Bowel Diseases*.

[11] Ghosh S. (2015). Inflammatory bowel disease: appreciating disability and impact of disease. *Canadian Journal of Gastroenterology and Hepatology*.

[12] Toward a common language for Functioning, disability and health. ICF. http://www.worldenablenet/escapstats/icfcommon.htm.

[13] Gower-Rousseau C., Sarter H., Savoye G. (2017). Validation of the inflammatory bowel disease Disability Index in a population-based cohort. *Gut*.

[14] Allen P. B., Kamm M. A., Peyrin-Biroulet L. (2013). Development and validation of a patient-reported disability measurement tool for patients with inflammatory bowel disease. *Alimentary Pharmacology & Therapeutics*.

[15] Üstün T. B., Kostanjsek N., Chatterji S. Measuring Health and disability: Manual for Disability Assessment Schedule (WHODAS 2.0) World Health Organization. http://www.who.int/classifications/icf/whodasii/en/.

[16] Israeli E., Graff LA., Clara I. (2014). Low prevalence of disability among patients with inflammatory bowel diseases a decade after diagnosis. *Clinical Gastroenterology and Hepatology*.

[17] Inflammatory bowel disease. World Gastroenterology Organisation Global Guidelines. http://www.worldgastroenterology.org/UserFiles/file/guidelines/inflammatory-bowel-disease-english-2015.pdf.

[18] Harvey R. F., Bradshaw J. M. (1980). A simple index of Crohn's-disease activity. *The Lancet*.

[19] Walmsley R. S., Ayres R. C. S., Pounder R. E., Allan R. N. (1998). A simple clinical colitis activity index. *Gut*.

[20] Pallis A. G., Vlachonikolis I. G., Mouzas I. A. (2001). Quality of life of Greek patients with inflammatory bowel disease. Validation of the Greek translation of the Inflammatory Bowel Disease Questionnaire. *Digestion*.

[21] Hawkey C. J., Allez M., Clark M. M. (2015). Autologous hematopoetic stem cell transplantation for refractory Crohn disease a randomized clinical trial. *JAMA*.

[22] Tsoukka M., Jelastopulu E., Lavranos G. (2017). Estimation of quality of life in Cypriot patients with inflammatory bowel disease. *World Journal of Gastroenterology*.

[23] Lesage A.-C., Hagège H., Tucat G., Gendre J.-P. (2011). Results of a national survey on quality of life in inflammatory bowel diseases. *Clinics and Research in Hepatology and Gastroenterology*.

[24] Muller K. R., Prosser R., Bampton P., Mountifield R., Andrews J. M. (2010). Female gender and surgery impair relationships, body image, and sexuality in inflammatory bowel disease: patient perceptions. *Inflammatory Bowel Diseases*.

[25] Becker H. M., Grigat D., Ghosh S. (2015). Living with inflammatory bowel disease: a Crohn's and Colitis Canada survey. *Canadian Journal of Gastroenterology and Hepatology*.

[26] Viazis N., Mantzaris G., Karmiris K. (2013). Inflammatory bowel disease: Greek patients' perspective on quality of life, information on the disease, work productivity and family support. *Annals of Gastroenterology*.

[27] Hendy P., Inspector Y., Hart A. (2015). Standard medical care, side effects and compliance. *Psychological Aspects of Inflammatory Bowel Disease: a Biopsychosocial Approach*.

[28] Rosenblatt E., Kane S. (2015). Sex-specific issues in inflammatory bowel disease. *Gastroenterology & Hepatology*.

[29] Longobardi T., Jacobs P., Bernstein C. N. (2003). Work losses related to Inflammatory bowel disease in the United States Folder: results from the National Health interview survey. *The American Journal of Gastroenterology*.

[30] Vester-Andersen M. K., Prosberg M. V., Vind I., Andersson M., Jess T., Bendtsen F. (2015). Low risk of unemployment, sick leave, and work disability among patients with inflammatory bowel disease: a 7-year follow-up study of a danish inception cohort. *Inflammatory Bowel Diseases*.

[31] Feagan B. G., Bala M., Yan S., Olson A., Hanauer S. (2005). Unemployment and disability in patients with moderately to severely active Crohn's disease. *Journal of Clinical Gastroenterology*.

[32] De Boer A. G. E. M., Evertsz F. B., Stokkers P. C. (2016). Employment status, difficulties at work and quality of life in inflammatory bowel disease patients. *European Journal of Gastroenterology and Hepatology*.

[33] Mandel M. D., Bálint A., Lovász B. D. (2014). Work disability and productivity loss in patients with inflammatory bowel diseases in Hungary in the era of biologics. *European Journal of Health Economics*.

[34] Argyriou K., Kapsoritakis A., Potamianos S. (2014). PTU-098high levels of emotional and physical distress among family caregivers of individuals with Inflammatory Bowel Disease (IBD). *Gut*.

[35] Devlen J., Beusterien K., Yen L., Ahmed A., Cheifetz A. S., Moss A. C. (2014). The burden of inflammatory bowel disease: a patient-reported qualitative analysis and development of a conceptual model. *Inflammatory Bowel Diseases*.

[36] Soares J. B., Pereira R., Costa J. M. (Oct 2016). The inflammatory bowel disease-disability index: validation of the Portuguese version according to the COSMIN checklist. *European Journal of Gastroenterology & Hepatology*.

[37] van der Have M., Fidder H. H., Leenders M. (2015). Self-reported disability in patients with inflammatory bowel disease largely determined by disease activity and illness perceptions. *Inflammatory Bowel Diseases*.

